# Curative Treatment of Prolapsus Uteri, by the Local Application of Tannin

**Published:** 1856-03

**Authors:** Charles A. Budd

**Affiliations:** Fellow of the N. Y. Academy of Medicine


*Curative Treatment of Prolapsus Uteri, by the local application of tannin.* By CHARLES A. BUDD, M. D., Fellow of the N. Y. Academy of Medicine.

The many and varied forms of uterine disease resulting from an injudicious use of pessaries, as well as the irreparable damage done by means of cauteries, either actual or potential, or by extirpation of portions of mucous membranes, which have for their object the diminution of the capacity af the vagina with a view of curing prolapsus, have led me to add my testimony in favor of a practice first recommended by Prof. B. F. Barker, of this city. I allude to the application of lint soaked in a saturated solution of tannin; and if the perusal of the following cases, selected from a variety which have come under my observation, will induce a trial of the treatment alluded to, I feel confident that in all cases to which it is applicable, the result will be equally gratifying. There are, to be sure, certain abnormal conditions in which it is not applicable, as, for example, a very straight sacrum, or a loss of substance in the perineum due to laceration or other causes; or if there be any pathological condition of the cervix, it must be removed by appropriate treatment; and then, if the prolapsus continue, the utility, I may say infallibility, of the method alluded to, becomes apparent. But in simple cases of prolapsus, whether incipient, partial, or complete, depending upon a mere relaxation of the uterine supports, I have never known it to fail where the treatment has been faithfully persevered in. The manner recommended by Prof. B., and the one I have followed, in its principal details, is as follows:—From a double thickness of lint a triangular portion is cut out, of a sufficient size to fill the capacity of the vagina when rolled up so as to form a cone, near the apex of which is attached a piece of string to facilitate withdrawal. The patient being placed upon her back, with the hips slightly elevated, the uterus is replaced in situ, and the lint, soaked in a saturated solution of tannin, is applied with its apex downward and its base immediately in contact with the os tincæ. This is repeated once in twenty-four hours, for a period of time in accordance with the extent of the displacement. I have usually found a daily application for a period of about a month, to be sufficient to perfect a cure. During this time, and subsequently, constipation must be rigidly guarded against, and the state of the general health attended to. The vagina soon begins to acquire its wonted tonicity and contractility, and the lint is consequently obliged gradually to be lessened in quantity; the strain being taken off the round ligaments, also allows them to return to their normal condition. The following cases exemplify the admirable effects of this plan of treatment.

CASE I.—Mrs. G., æt. 24 years, the mother of two children, the youngest fifteen months of age, had always enjoyed good health until the birth of her last child. The placenta in this last accouchement (I having attended her in both) was retained over three hours, in consequence of irregular uterine contractions. She has been complaining ever since of pain in the lumbo-sacral region, with bearing-down pains in the hypogastrium; great constipation, with vesical and rectal tenesmus; and a sensation of faintness after an evacuation, leucorrhœa, &c. &c. Upon examination, I found the cervix protruding at the vulva, extensive ulceration extending into the canal of the cervix, inflammation of the posterior wall of the uterus, and enlargement of the organ itself, its long diameter measuring 4½ inches. After three months' treatment, these conditions, save the prolapsus, were all removed, the applications to the cervix having been made weekly, and without the aid of a speculum, and the uterus at this time measuring less than three inches in its long axis. The treatment consisting of the lint and tannin, was soon after commenced; and in about three weeks' time she was enabled to resume her ordinary duties. She is now four months pregnant with her third child, all treatment having been suspended about six months ago.

Case II.—Mrs. S., æt. 20 years, the mother of one child aged two years applied to me in July, 1854, suffering from all the symptoms of uterine prolapsus. She had aborted with a three months' fœtus about a month previous, but had been complaining for two years before. An examination revealed incipient prolapsus, the cervix lying on the floor of the perineum, and slight epithelial abrasion of its mucous surface. Two applications of nitrate of silver removed this; and the use, for ten days, of the lint and tannin, effected a perfect cure.

Case III.—Mrs. G., æt. 54 years. A widow, the mother of five children, the youngest sixteen years of age. Had ceased menstruating about ten years previous. She stated to me that upon using the slightest exertion, such as lifting or straining at defœcation, her womb would entirely protrude from the vulva. She had used a variety of abdominal supporters, and had attempted on several occasions to wear pessaries of different kinds, and at that time was wearing constantly a T bandage. Upon examination I found the uterus just within the vulva; and, requesting her to lift a chair, the whole organ was protruded, dragging with it the posterior wall of the bladder; it was perfectly healthy in appearance, though somewhat atrophied. I commenced the treatment with the lint and tannin, interdicting active exercise, and in six weeks ceased making any applications. She gradually resumed her ordinary duties, and is now (some two years since) perfectly recovered, and is considered a very active old lady. She has not had the slightest disposition to a return of the displacement, and enjoys excellent health.

I have here given an example of the three different degrees of prolapsus,—incipient, partial and complete,—illustrating the curability of this treatment in each. I could, if it were desirable, cite many others which have been under my observation, and which have resulted, without a single exception, in a perfect and complete restoration.—*New York Medical Times,* Feb. 1856.

